# Research hotspots and trends on post-cesarean section analgesia: A scientometric analysis from 2001 to 2021

**DOI:** 10.1097/MD.0000000000034973

**Published:** 2023-10-06

**Authors:** Ziwei Zhao, Zhongbiao Nie, Yanyan Li, Peili Wang, Ran Zhang

**Affiliations:** a Affiliated Hospital of Shanxi University of Chinese Medicine, Taiyuan, China; b Shanxi Bethune Hospital, Shanxi Academy of Medical Sciences, Tongji Shanxi Hospital, Third Hospital of Shanxi Medical University, Taiyuan, China; c Shanxi University of Chinese Medicine, Jinzhong, China; d Xiyuan Hospital, China Academy of Chinese Medical Sciences, Beijing, China.

**Keywords:** analgesia, Citespace, knowledge mapping, post-cesarean section, scientometric analysis

## Abstract

This study aims to demonstrate current research priorities and predict future trends of post-cesarean section analgesia by scientometric analysis. We collected nearly 20 years (2002–2021) of publications related to post-cesarean section analgesia in the web of science database. Citespace was applied to evaluate the knowledge mapping. There are 2735 manuscripts about the post-cesarean section in total. The country, institution, and author posted the most separately are the USA, Univ Calif Irvine, and BRENDAN CARVALHO. INTERNATIONAL JOURNAL OF OBSTETRIC ANESTHESIA (21) publishes the most articles of this type, and ANESTHESIOLOGY has the greatest impact (1496 co-citations). In addition, the most key cited reference is McDonnell, J.G (43). Post-cesarean section analgesia research, including spinal anesthesia, postoperative pain, and epidural analgesia, has been a research hotspot in recent years. Through scientometric analysis of the past 20 years, we know the TAP blocks and drug selection in patient-controlled analgesia are the focus of future research. The USA, China, and Turkey have become the main research forces in this field, with high publication rates and centrality. This is important for accurately and quickly locating trends in this field.

## 1. Introduction

Different levels of pain, from moderate to severe, usually occurs after the post-cesarean section. Poor pain may reduce milk production, limit women normal activities, and increase the risk of thromboembolism. Therefore, good postpartum analgesia is very important.^[1–4]^ In recent years, there have been more and more studies on postoperative analgesia for post-cesarean section patients, including surgical mode, analgesia mode, and drug selection. It is essential to grasp the research trends of post-cesarean section analgesia. Due to the rapid growth of cesarean section analgesia research, it is a challenge to entirely understand its research status and hotspots.

Scientometrics is a quantitative analysis of a specific discipline. Including the different database: Pubmed,^[5]^ Web of science (WOS),^[6]^ Scopus,^[7]^ Derwent,^[8]^ etc. Among them, WOS is analyzed by different software (Histcite,^[9]^ Citespace,^[8]^ VOSviewer,^[10]^ etc) that can be used for scientometric analysis. Readers can completely understand the hotspots, trends, and frontiers in this field through Citspace software, which helps us to illustrate the development background of certain research areas.^[11]^ Citespace is a mature visualization software.^[12]^ It explores the key point in the evolution of subject fields. It includes coauthor, co-citation, and co-occurrence analysis,^[13]^ and 3 concepts burst detection, betweenness centrality, and heterogeneous network.^[14]^

There is currently no bibliometric method to study the analgesia of the post-cesarean section. Therefore, in this literature, researchers used Citespace software to analyze the global role and trend of post-cesarean section analgesia in the WOS database, from January 1, 2001 to December 20, 2021, and established a field knowledge map in this study.

## 2. Method

### 2.1. Source of literature

We input the WOS database with subject words: TS = (postoperative pain * OR analgesia * OR analgesic * OR obstetrical * OR Patient-Controlled * OR after operative pain) AND TS = (cesarean * OR cesarean section * OR cesarean delivery). The search scope of the database covers from January 1, 2001 to December 20, 2021, and the language type was English. Through a literature search, 3211 records were gained. After Citespace eliminated duplication, 2735 pieces of literature were used for quantitative analysis. The WOS database comes from the Shanxi Bethune Hospital database in China.

### 2.2. Analysis software

Citespace analysis software version is Version 5.6. R2.^[13]^

### 2.3. Download and import of data

Export the results of the retrieved subject terms, and keep the file format as “plain text.”

### 2.4. Parameter setting

Time slicing (from 2001 to 2021; node type (check one at a time); selection criteria (50); pruning (pathfinder); visualization (show merged network, cluster view-static).

### 2.5. Statistical methods

**Figure 1.**
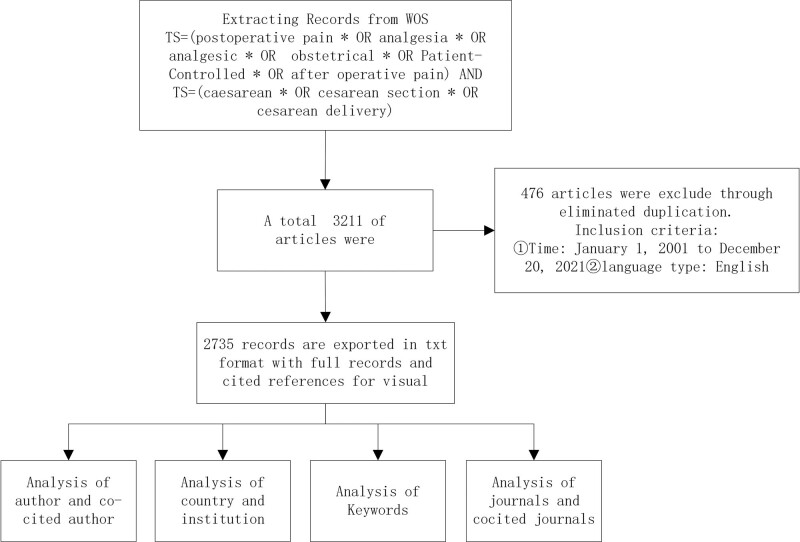
Analysis flow chart of post-cesarean section analgesia.

All literature have been scientifically analyzed; Some data obtained include core countries, institutions, authors, keywords, and references.^[14–16]^ The detailed analysis flow is shown in Figure 1

## 3. Results

### 3.1. Analysis of countries and institutions

**Figure 2.**
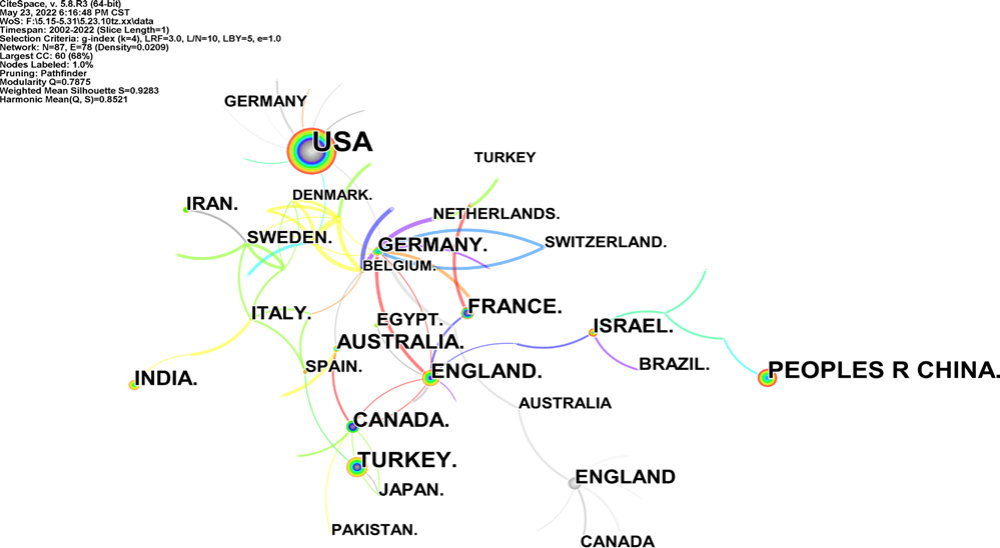
Analysis of the country map from 2001 to 2021.

A country map was generated (Fig. 2). 88 countries published 2735 references. The USA, Peoples R China, Turkey, England, and Canada are the top 5 countries (Table 1). Chile. (0.49) and the USA (0.42) were the top 2 countries from centrality (purple round). Analysis of the number of publication and centrality shows that the USA, People R China, and Turkey were the main research force in post-cesarean section analgesia. Australia, France, and India have continued to increase their research interest in this field. The research is mainly distributed in developed countries, and the cooperation between countries is weak.

**Table 1:** Top 10 countries and institutions researching post-cesarean section analgesia.

Ranking	Country	Publications	Institution	Publications
1	USA	654	Stanford Univ	62
2	Peoples R China	230	Duke Univ	34
3	Turkey	138	Harvard Med Sch	31
4	England	110	Karolinska Inst	29
5	Canada	98	Univ Toronto	26
6	Australia	93	Tel Aviv Univ	24
7	India	92	KK Womens & Childrens Hosp	22
8	France	92	Northwestern Univ	21
9	Israel	79	Columbia Univ	21
10	England	77	Univ British Columbia	20

**Figure 3.**
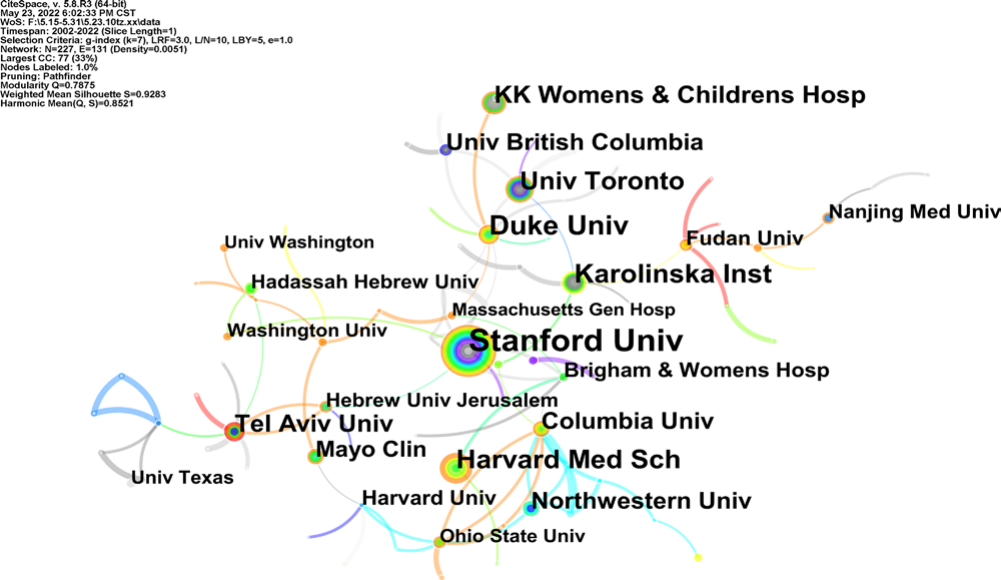
Analysis of the institutional map from 2001 to 2021.

An institution map with 227 nodes and 131 links was generated (Fig. 3). The 2735 publications have been published in 227 research institutions. Stanford Univ, Duke Univ, Harvard Med Sch, Karolinska Inst, and Univ Toronto are the top 5 institutions (Table 1). In terms of centrality, the top 3 institutions were Karolinska Inst (0.08), Columbia Univ (0.07), and Duke Univ (0.06). In addition, the links between institutions are relatively thin, indicating that cooperation is weak.

### 3.2. Analysis of journals and co-cited journals

The top 10 academic journals related to post-cesarean section analgesia see in the Table 2. They are specialized journals in this field.

**Table 2:** Top 10 academic journals related to research post-cesarean section analgesia.

Ranking	Journal	Publications	IF (2021)
1	INTERNATIONAL JOURNAL OF OBSTETRIC ANESTHESIA	21	3.282
2	ANESTHESIA AND ANALGESIA	21	6.627
3	JOURNAL OF CLINICAL ANESTHESIA	20	9.375
4	REGIONAL ANESTHESIA AND PAIN MEDICINE	19	5.564
5	OBSTETRICS AND GYNECOLOGY	19	7.623
6	INTERNATIONAL JOURNAL OF GYNECOLOGY & OBSTETRICS	19	4.447
7	ACTA ANAESTHESIOLOGICA SCANDINAVICA	19	2.274
8	ANESTHESIOLOGY	19	8.986
9	EUROPEAN JOURNAL OF ANAESTHESIOLOGY	18	4.183
10	JOURNAL OF MATERNAL-FETAL & NEONATAL MEDICINE,	18	2.323

IF = impact factor.

Some articles are highly cited, such as An Updated Report by the American Society of Anesthesiologists Task Force on Obstetric Anesthesia and the Society for Obstetric Anesthesia and Perinatology^[17]^ These guidelines are intended to improve the level of anesthesia and analgesia of maternal and to improve patient safety and satisfaction.

**Figure 4.**
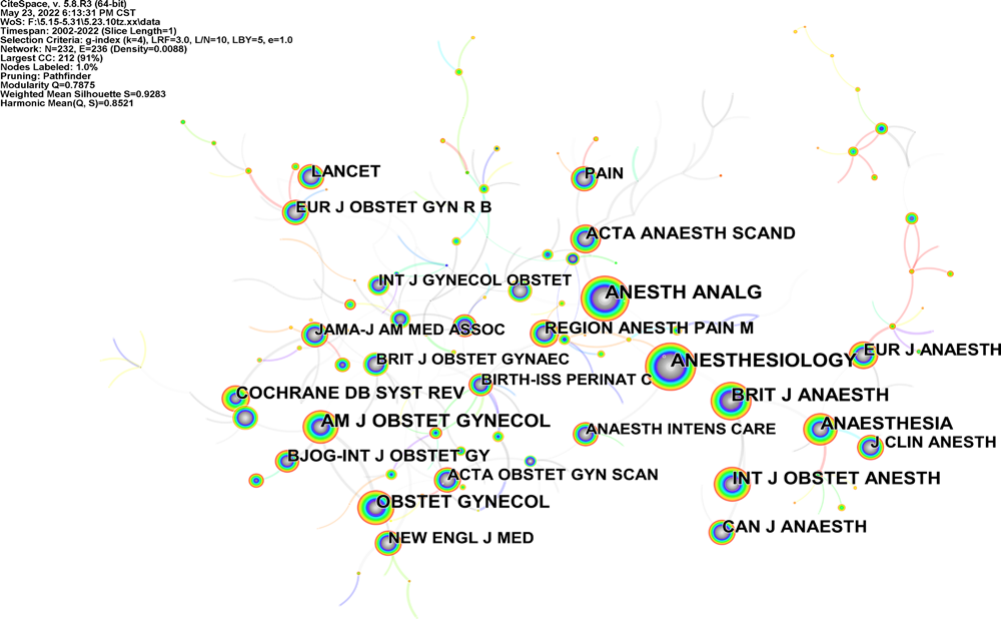
Analysis of the co-cited journal map from 2001 to 2021.

A co-cited journal map that had 232 nodes and 236 links was generated (Fig. 4). ANESTH ANALG, ANESTHESIOLOGY, BRIT J ANAESTH, ANESTHESIOLOGY, AM J OBSTET GYNECOL are the top 5 co-cited journals, ANESTHESIOLOGY, BRIT J ANAESTH, ANESTHESIOLOGY, ACTA OBSTET GYN, ANESTHESIOLOGY are the top 5 centralities (Table 3, Fig. 3). In an analysis of number of publication and centrality, the core journal is the ANESTHESIOLOGY, which was related to the topic of analgesia in our study. It published pieces of literature reflect the current state of research in this field.

**Table 3:** Top 10 co-cited journals and centrality related to post-cesarean section analgesia research from 2001 to 2021.

Ranking	Co-citation counts	Cited journal	Ranking	Centrality	Cited journal
1	1631	ANESTH ANALG	1	0.56	ANESTHESIOLOGY
2	1496	ANESTHESIOLOGY	2	0.51	BRIT J ANAESTH
3	1356	BRIT J ANAESTH	3	0.5	ACTA OBSTET GYN SCAN
4	1097	AM J OBSTET GYNECOL	4	0.46	ANAESTHESIA
5	1090	INT J OBSTET ANESTH	5	0.45	BIRTH-ISS PERINAT C
6	1069	OBSTET GYNECOL	6	0.41	BRIT J OBSTET GYNAEC
7	972	ANAESTHESIA	7	0.37	ANESTH ANALG
8	803	ACTA ANAESTH SCAND	8	0.37	CLIN PHARMACOL THER
9	689	COCHRANE DB SYST REV	9	0.36	J REPROD MED
10	676	CAN J ANAESTH	10	0.35	PEDIATRICS

### 3.3. Analysis of author and co-cited author

**Figure 5.**
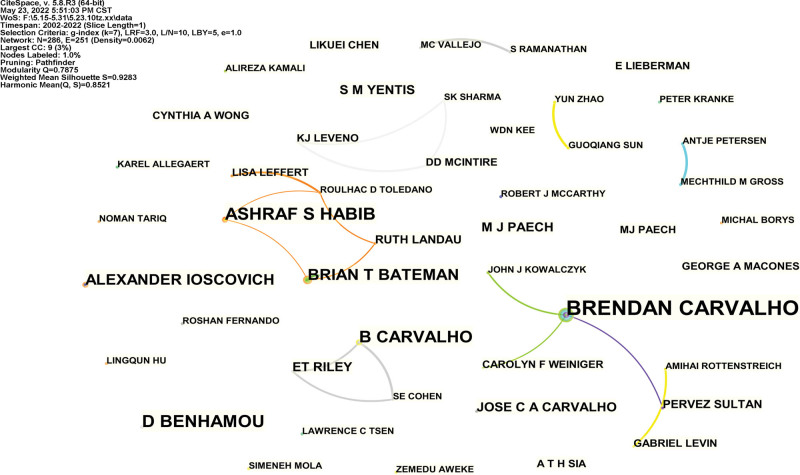
Analysis of the coauthor map from 2001 to 2021.

**Figure 6.**
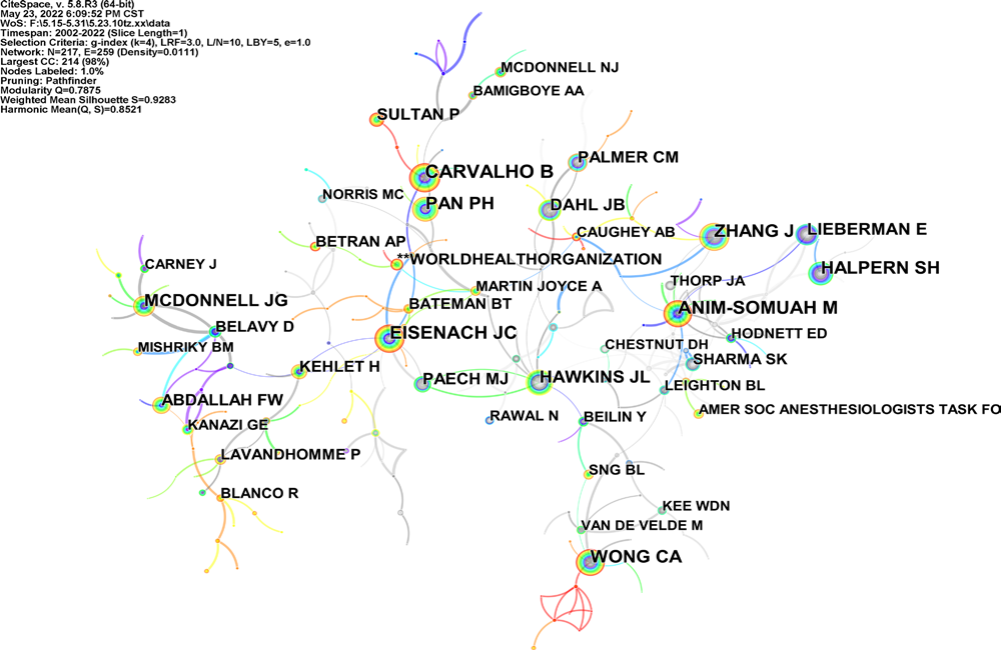
Analysis of the co-cited author map from 2001 to 2021.

286 research authors published totally 2600 articles. The top ten authors with published articles about post-cesarean section analgesia are the experts of this field (Table 4). A coauthor map that had 286 nodes and 251 links was generated (Fig. 5). A co-cited author map that had 217 nodes and 259 links was generated (Fig. 6). CARVALHO B, EISENACH JC, ANIM-SOMUAH M, PAN PH, WONG CA are the top 5 co-cited authors, and HAWKINS JL, SHARMA SK, EISENACH JC were the top 3 centralities (Table 5, Fig. 6).

**Table 4:** Top 10 authors in post-cesarean section analgesia research from 2001 to 2021.

Ranking	Author	Publications
1	BRENDAN CARVALHO	25
2	ASHRAF S HABIB	13
3	D BENHAMOU	13
4	B CARVALHO	12
5	BRIAN T BATEMAN	10
6	ALEXANDER IOSCOVICH	9
7	JOSE C A CARVALHO	6
8	S M YENTIS	6
9	ET RILEY	6
10	M J PAECH	6

**Table 5:** Top 10 co-cited authors in post-cesarean section analgesia research in terms of co-citation and centrality.

Ranking	Author	Co-citation counts	Centrality	Author
1	CARVALHO B	198	HAWKINS JL	1.08
2	EISENACH JC	175	EISENACH JC	0.8
3	ANIM-SOMUAH M	157	SHARMA SK	0.76
4	PAN PH	142	PAECH MJ	0.61
5	WONG CA	141	KEHLET H	0.61
6	ZHANG J	140	BEILIN Y	47
7	HALPERN SH	126	COSTELLO JF	33
8	MCDONNELL JG	123	ZHANG J	140
9	HAWKINS JL	112	THORP JA	54
10	LIEBERMAN E	103	FRIEDMAN EA	5

As the author with the most published and co-cited articles, BRENDAN CARVALHO is based at the Stanford University School of Medicine, and their team has made a detailed study on cesarean delivery analgesia. He experimentally confirmed that intrathecal morphine, in combination with fentanyl, is an effective and safe cesarean section analgesic that can replace dimorphine.^[18]^ Diamorphine has also gradually withdrawn from this stage of history. One of his meta-analyses found that enhanced recovery after cesarean delivery (ERAC) implementation is associated with reduced postoperative opioid consumption.^[19]^ In order to implement ERAC pathways for obstetric patients, hospital systems must have highly functional interactions with regional cultural elements. The patient experience is improved through ERAC pathways, which also support a community growth orientation, dedication to continuous improvement, and high standards of clinical care.^[20]^ His research has contributed greatly to post-cesarean section analgesia. An analysis by centrality and cocitation counts revealed that EISENACH JC and HAWKINS JL were “core strength” researchers. They have had an important impact on research in this area. EISENACH JC is likely to continue production in this area in the future.

### 3.4. Analysis of co-cited references

The reference co-cited map that had 287 nodes and 327 links was generated (see Fig. S1, Supplemental Fig. S1, http://links.lww.com/MD/J683, which demonstrates keywords map). An analysis of counts and centrality (see Tables S1, S2. Supplemental Table S1, http://links.lww.com/MD/J684, which demonstrates top 5 co-cited references in terms of co-citation counts. Supplemental Table S2, http://links.lww.com/MD/J685, which demonstrates top 5 co-cited references in terms of centrality) revealed that the data usually comes in the form of random trials. Among them, “Practice Guidelines for Obstetric Anesthesia (2016)” (impact factor [IF]: 8.986) was published in Anesthesiology in 2016. This guideline was reported and updated by the American society of anesthesiologists. The American society of anesthesiologists is an educational, researchful, and scientific physicians’ association, organized to raise the standards of the medical practice in anesthesiology and to improve patients’ nursing. It believed that for postoperative analgesia after nerve axis anesthesia in cesarean section, consideration should be given to choosing a neuraxial opioid rather than intermittent parenteral opioids. Guidelines are the most cited and illustrate the standardization of clinical research and treatment.

### 3.5. Analysis of keywords

Figure S2 (see Fig. S2, Supplemental Fig. S2, http://links.lww.com/MD/J686, which demonstrates the co-occurrence network of keywords map) shows the occurrence network of keywords. The analysis divided them into 4 clusters: cluster 1 (spinal anesthesia research, red); Cluster 2 (postoperative pain research, yellow); Cluster 3 (epidural analgesia research, canary yellow); Cluster 4 (patient-controlled analgesia, green). In cluster 1, high rates were “spinal anesthesia (59.4, 1.0E^−4^); section (54.72, 1.0E^−4^); efficacy (39.35, 1.0E^−4^).” In cluster 2, the high rate keywords were “postoperative pain (52.99, 1.0E^−4^), general anesthesia (20.76, 1.0E^−4^), analgesics opioid (16.94, 1.0E^−4^).” In cluster 3, the high rate was “epidural analgesia (96.68, 1.0E^−4^); cesarean delivery (79.45, 1.0E^−4^); post-cesarean section (32.72, 1.0E^−4^), and in cluster 4, the high rate keywords were “post-cesarean section (77.41, 1.0E^−4^); bupivacaine (43.88, 1.0E^−4^); anesthesia (38.05, 1.0E^−4^).

Keywords can reflect the hotspots and research directions in the field of research to a certain extent. From these keywords, we can know that local anesthetics may become the focus of research in the future.

## 4. Discussion

By analyzing references and keywords, we found that spinal epidural anesthesia was the main modality of cesarean section, and patient-controlled analgesia (PCA) was the main mode of it. Studies have shown that epidural analgesia may be more effective than non-epidural analgesia in reducing postoperative pain during labor and increasing maternal satisfaction with pain relief.^[21]^ PCA is a common method of relieving postoperative pain in hospitalized patients, by titrating painkillers on demand. It generally provides better pain control and improved patient satisfaction than “on-demand” opioid injections,^[22–24]^ so it has become a recent research hotspot.

By analyzing co-cited references, we found that the main research contents in the field of post-cesarean section analgesia are: the analgesic effect of transversus abdominis plane (TAP) block, the analgesic effect of multimodal analgesia and the combination of optimal analgesic drugs.^[22]^ Thanks to the concept of multimodal analgesia pain management^[25]^ as an important component of opioid analgesia regimens, several meta-analyses have shown that the use of TAP blocks provides good analgesia and reduces opioid consumption immediately after surgery.^[26]^ Studies showed that cesarean section is a type of surgery that is suitable for the TAP,^[27]^ because the traditional Pfannenstiel incision is located in an area that is prone to lateral approach anesthesia that is usually performed. To date, Caesarean section is the most studied transverse abdominal plane block.^[28,29]^ Regarding the choice of drugs, many randomized controlled trials have concluded that a drug program consisting of acetaminophen, nonsteroidal anti-inflammatory drugs significantly relieves pain and improve the patient experience.^[30,31]^

Research cluster has been selected as the main research topic in this field. In a cluster, the main keywords were “spinal anesthesia,” “postoperative pain,” and “epidural analgesia.” To date, we found that spinal anesthesia and epidural analgesia are still the main way of analgesia after cesarean section at present. At present, spinal anesthesia and epidural injection of opioids are the 2 main forms of analgesia for the post-cesarean section. Most recent studies have focused on the choice of analgesic drugs. The most studied drugs include hydromorphone,^[32]^ sufentanil,^[22]^ morphine.^[18]^ A meta-analysis found that PCA with hydromorphone is more effective in relieving pain and PCA requests after the operation and significantly reduced the incidence of adverse events than sufentanil.^[33]^ But new opioids appear to be most used in China.

After a cesarean section, receiving adequate pain management has several benefits for the mother, the baby, as well as society. The PROSPECT evidence-based recommendations emphasize the importance of multimodal analgesic approach as a means to provide high-quality, effective pain management.^[34]^ Highly effective pain management is achieved by combining systemic medications (scheduled paracetamol, scheduled nonsteroidal anti-inflammatory agents, and dexamethasone), local methods (intrathecal morphine or alternatively either wound infiltration/infusion, a TAP block, or a QL block), and surgical procedures (abdominal binders, non-closure of the peritoneum, and a Joel-Cohen incision). Recent auditing of the PROSPECT guideline careful application in a sizable tertiary care teaching hospital revealed good pain scores with little requirement for rescue opioid analgesia.^[35,36]^

## 5. Conclusion

Bibliometric analysis of post-cesarean section analgesia publications from 2001 to 2021 revealed that the TAP blocks and drug selection in PCA are the focus of future research. The USA, China, and Turkey have become the main research forces in this field, with high publication rates and centrality. Many developed countries have the strongest cooperation with well-known institutions, which is conducive to the development of post-cesarean section analgesia research. These articles are widely cited because they are guidelines or highly IF.

## 6. Limitations

This technique may have missed publications published in other databases since we solely used CiteSpace software for visual examination of the WOS database in this investigation. Additionally, the search technique was developed to gather as much information as possible, therefore it cannot be assumed that all included articles are entirely pertinent to the subject of the study.

## Acknowledgments

The authors would like to express their appreciation to Professor Chaomei Chen for inventing Citespace and making it free to use.

## Author contributions

**Conceptualization:** Ziwei Zhao, Zhongbiao Nie, Peili Wang.

**Data curation:** Zhongbiao Nie.

**Funding acquisition:** Ran Zhang.

**Investigation:** Ziwei Zhao, Ran Zhang.

**Methodology:** Yanyan Li, Ran Zhang.

**Project administration:** Ziwei Zhao, Yanyan Li, Ran Zhang.

**Resources:** Ziwei Zhao, Ran Zhang.

**Software:** Zhongbiao Nie, Yanyan Li.

**Supervision:** Ziwei Zhao, Zhongbiao Nie, Yanyan Li, Peili Wang.

**Visualization:** Zhongbiao Nie.

**Writing – review & editing:** Zhongbiao Nie.

**Writing – original draft:** Peili Wang.

## Supplementary Material
