# Supplementary figures and images for: Research hotspots and trends on post-cesarean section analgesia: A scientometric analysis from 2001 to 2021
Source: Medicine (Baltimore). 2023 Oct 6;102(40):e34973. doi: 10.1097/MD.0000000000034973 (PMC10553133; doi:10.1097/MD.0000000000034973)

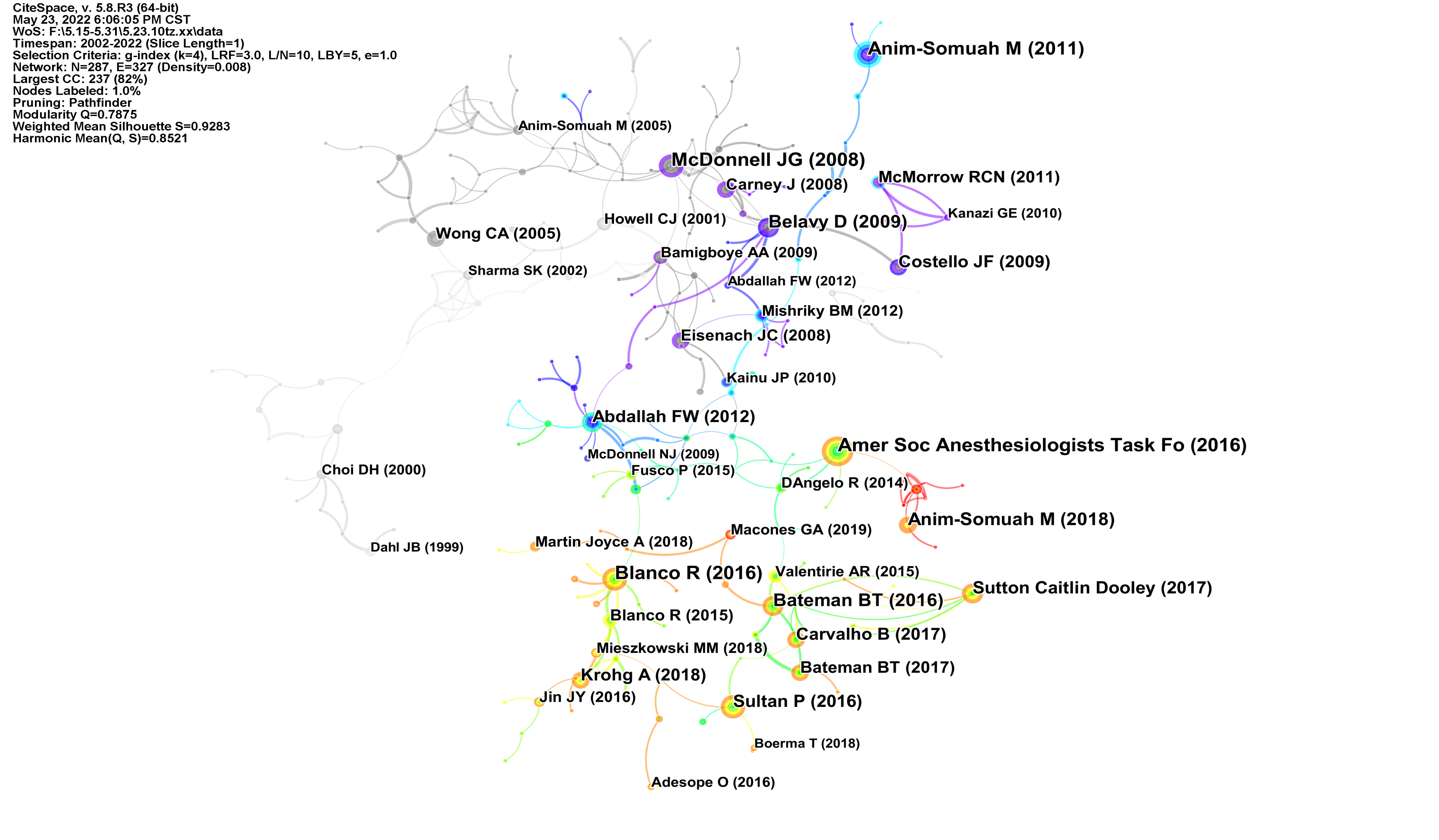

Supplement: Supplementary file 1 [file medi-102-e34973-s001.tiff]

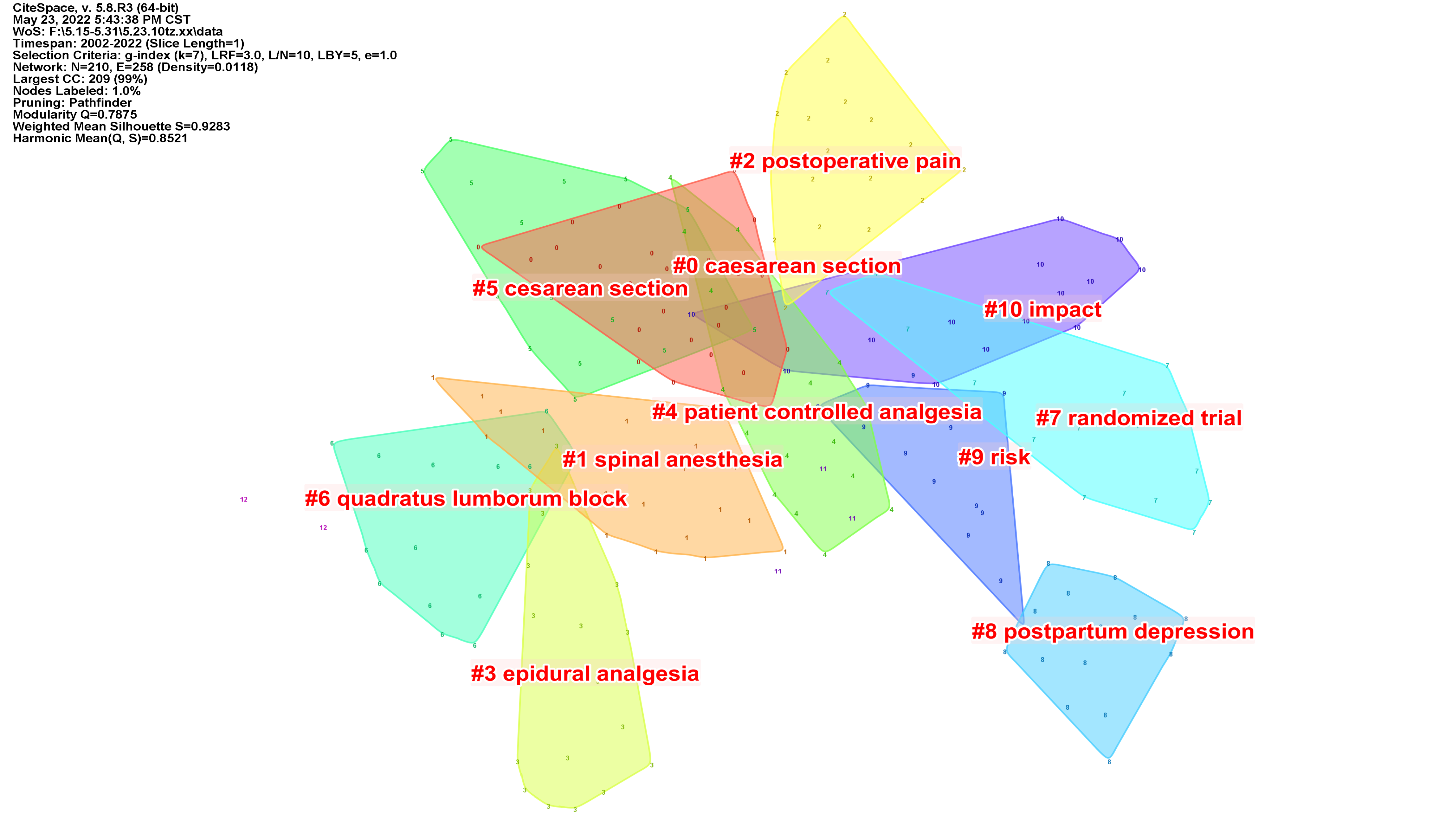

Supplement: Supplementary file 4 [file medi-102-e34973-s004.tiff]
