# Supplementary material for: Research hotspots and trends on post-cesarean section analgesia: A scientometric analysis from 2001 to 2021
Source: Medicine (Baltimore). 2023 Oct 6;102(40):e34973. doi: 10.1097/MD.0000000000034973 (PMC10553133; doi:10.1097/MD.0000000000034973)
Supplement: Supplementary file 2 [file medi-102-e34973-s002.docx]

**Table S1** Top five co-cited references related to post-cesarean section analgesia research in terms of co-citation counts

| **Ranking** | **Cited reference** | **Co-citation counts** | **Representative author (publication year)** |
| --- | --- | --- | --- |
| **1** | Practice Guidelines for Obstetric Anesthesia^[1]^ | 45 | Faiz S(2016) |
| **2** | The Analgesic Efficacy of Transversus Abdominis Plane Block After Cesarean Delivery: A Randomized Controlled Trial^[2]^ | 43 | McDonnell J G(2008) |
| **3** | Quadratus Lumborum Block Versus Transversus Abdominis Plane Block for Postoperative Pain After Cesarean Delivery: A Randomized Controlled Trial^[3]^ | 43 | Blanco R(2016) |
| **4** | Epidural versus non-epidural or no analgesia in labour^[4]^ | 42 | Anim-Somuah M(2011) |
| **5** | Epidural versus non-epidural or no analgesia for pain management in labour^[5]^ | 37 | Anim-Somuah M(2018) |

**Reference**

[1] Faiz S, Alebouyeh M R, Derakhshan P, et al. Comparison of ultrasound-guided posterior transversus abdominis plane block and lateral transversus abdominis plane block for postoperative pain management in patients undergoing cesarean section: a randomized double-blind clinical trial study[J]. J Pain Res, 2018,11:5-9.

[2] McDonnell J G, Curley G, Carney J, et al. The analgesic efficacy of transversus abdominis plane block after cesarean delivery: a randomized controlled trial[J]. Anesth Analg, 2008,106(1):186-191.

[3] Blanco R, Ansari T, Riad W, et al. Quadratus Lumborum Block Versus Transversus Abdominis Plane Block for Postoperative Pain After Cesarean Delivery: A Randomized Controlled Trial[J]. Reg Anesth Pain Med, 2016,41(6):757-762.

[4] Anim-Somuah M, Smyth R M, Jones L. Epidural versus non-epidural or no analgesia in labour[J]. Cochrane Database Syst Rev, 2011(12):CD331.

[5] Anim-Somuah M, Smyth R M, Cyna A M, et al. Epidural versus non-epidural or no analgesia for pain management in labour[J]. Cochrane Database Syst Rev, 2018,5:CD331.
