# Supplementary material for: Research hotspots and trends on post-cesarean section analgesia: A scientometric analysis from 2001 to 2021
Source: Medicine (Baltimore). 2023 Oct 6;102(40):e34973. doi: 10.1097/MD.0000000000034973 (PMC10553133; doi:10.1097/MD.0000000000034973)
Supplement: Supplementary file 3 [file medi-102-e34973-s003.docx]

**Table S2** Top five co-cited references in post-cesarean section analgesia research in terms of centrality

| **Ranking** | **Cited reference** | **centrality** | **Representative author (publication year)** |
| --- | --- | --- | --- |
| 1 | The Analgesic Efficacy of Transversus Abdominis Plane Block After Cesarean Delivery: A Randomized Controlled Trial^[1]^ | 0.74 | McDonnell J G(2008) |
| 2 | Transversus abdominis plane block for postoperative analgesia after Caesarean delivery performed under spinal anaesthesia? A systematic review and meta-analysis^[2]^ | 0.74 | Abdallah F W(2012) |
| 3 | Ultrasound-guided transversus abdominis plane block for analgesia after Caesarean delivery^[3]^ | 0.73 | Belavy D(2009) |
| 4 | Plasma ropivacaine concentrations after ultrasound-guided transversus abdominis plane block^[4]^ | 0.68 | Griffiths J D(2010) |
| 5 | A randomised trial of the analgesic efficacy of ultrasound-guided transversus abdominis plane block after caesarean delivery under general anaesthesia^[5]^ | 0.68 | Tan T T(2012) |

**Reference**

[1] McDonnell J G, Curley G, Carney J, et al. The analgesic efficacy of transversus abdominis plane block after cesarean delivery: a randomized controlled trial[J]. Anesth Analg, 2008,106(1):186-191.

[2] Abdallah F W, Halpern S H, Margarido C B. Transversus abdominis plane block for postoperative analgesia after Caesarean delivery performed under spinal anaesthesia? A systematic review and meta-analysis[J]. Br J Anaesth, 2012,109(5):679-687.

[3] Belavy D, Cowlishaw P J, Howes M, et al. Ultrasound-guided transversus abdominis plane block for analgesia after Caesarean delivery[J]. Br J Anaesth, 2009,103(5):726-730.

[4] Griffiths J D, Barron F A, Grant S, et al. Plasma ropivacaine concentrations after ultrasound-guided transversus abdominis plane block[J]. Br J Anaesth, 2010,105(6):853-856.

[5] Tan T T, Teoh W H, Woo D C, et al. A randomised trial of the analgesic efficacy of ultrasound-guided transversus abdominis plane block after caesarean delivery under general anaesthesia[J]. Eur J Anaesthesiol, 2012,29(2):88-94.

**校对报告**

当前使用的样式是 [中华人民共和国国家标准_GBT_7714-2005]

当前文档题录总数为5条，在5个位置共计插入5次（包括重复插入）

有0条题录存在必填字段内容缺失的问题

所有题录的数据正常
